# Association of Infant Feeding Practices with Iron Status and Hematologic Parameters in 6-Month-Old Infants

**DOI:** 10.3390/children8121159

**Published:** 2021-12-08

**Authors:** Chayatat Ruangkit, Nawapat Prachakittikul, Nutthida Hemprachitchai, Oraporn Dumrongwongsiri, Sasivimon Soonsawad

**Affiliations:** 1 Ramathibodi Medical School, Chakri Naruebodindra Medical Institute, Faculty of Medicine Ramathibodi Hospital, Mahidol University, Bang Phli 10540, Samut Prakan, Thailand; chayatat.rua@mahidol.ac.th; 2 Ramathibodi Chakri Naruebodindra Hospital, Chakri Naruebodindra Medical Institute, Faculty of Medicine Ramathibodi Hospital, Mahidol University, Bang Phli 10540, Samut Prakan, Thailand; nawapat.prc@student.mahidol.ac.th (N.P.); nutthida.hep@student.mahidol.ac.th (N.H.); 3 Department of Pediatrics, Faculty of Medicine Ramathibodi Hospital, Mahidol University, Ratchatewi 10400, Bangkok, Thailand; oraporn.roj@mahidol.ac.th

**Keywords:** infant feeding, breastfeeding, formula feeding, iron supplements, iron deficiency anemia

## Abstract

Background: Infants’ feeding practices in the first 6 months of life and their association with iron status and hematologic parameters has not been well studied. We aim to evaluate this association. Methods: In a retrospective chart review, we identified 403 infants who received laboratory screening for anemia at 6-month visits. Infants were categorized into four groups according to feeding practices. Hematologic parameters and incidence of anemia, iron deficiency (ID), and iron deficiency anemia (IDA) were compared. Results: In total, 105 infants were breastfed (BF), 78 were breastfed with iron supplementation starting at 4 months (BI), 109 were mixed-fed (breast milk and formula) with or without iron supplementation (MF), and 111 were formula-fed (FF). The BF group had the highest incidence of anemia (38.1%), ID (28.6%), and IDA (17.1%) when compared with the other groups (*p* < 0.001). In multivariate logistic regression, BI, MF, and FF infants had 90.4%, 97.5%, and 96.9% decreased risk of IDA, respectively, with BF infants as a reference group. Conclusion: The incidence of anemia, ID, and IDA at age 6 months was higher in BF than FF or MF infants. However, iron supplements in BF infants starting at 4 months significantly reduced their ID and IDA incidence.

## 1. Background

Iron deficiency (ID), one of the most common nutritional deficiencies, remains a significant global public health challenge in developed and developing countries [1]. Because iron is an essential component of hemoglobin, the main component of red blood cells, ID is the most common cause of anemia worldwide [2,3]. Iron deficiency anemia (IDA) deleteriously affects many body systems and organ functions [4]. Among the most concerning detrimental effects of IDA in children are those involving behavior, cognition, and psychomotor skills. Several previous studies have indicated an association between ID in infants and poor neurodevelopmental outcomes [5,6].

A full-term infant has iron stores at birth that are sufficient until 4–6 months of age. During the first 6 months of life, infant iron status depends more on the iron store at birth than on the iron intake from breast milk. The iron content in breast milk is low and decreases over the lactation period. Nevertheless, because breast milk is considered the best nutrition for infants during the first year of life, especially in the first 6 months, the World Health Organization (WHO) and United Nations Children’s Fund (UNICEF) promote breastfeeding as a global public health policy [7]. In Thailand, The Royal College of Pediatricians of Thailand & Pediatric Society of Thailand and the Ministry of Public Health recommend that infants under 6 months of age be exclusively breastfed, with no need for complementary feeding or vitamin and mineral supplementation [8,9]. Previous studies on iron status in infants have shown that anemia and IDA occur more frequently in breastfed infants than in those who are formula-fed [10,11]. A cross-sectional study of iron status in breastfed infants aged 3–5 months showed a higher prevalence of ID and IDA among 5-month-old compared with 3-month-old infants [12]. A longitudinal study following the iron status of healthy breastfed infants at 4 and 6 months of age showed an increase in the prevalence of ID (5.7% to 26.1%) and IDA (3.4% to 23.9%) among infants at 4 months compared with those at 6 months [13]. These findings raise questions regarding iron adequacy among breastfed infants during the first 4–6 months of life. Consequently, although fully supporting breastfeeding, the American Academy of Pediatrics (AAP) recommended in 2010 that exclusively breastfed infants be given 1 mg/kg/day iron supplementation after 4 months of age to prevent IDA [14]. However, the risks and benefits of this practice remain inconclusive.

To our knowledge, there is limited information regarding iron status among Thai infants during the first 6 months of life, which is the age before iron-rich complementary feeding or iron supplementation should be introduced, as recommended by many health organizations. However, because The Royal College of Pediatricians of Thailand and Pediatric Society of Thailand had suggested universal screening for anemia among infants between age 6 and 12 months [15], many studies have investigated the iron status of infants during this period. The findings show that 34.0–62.5% of infants have serum ferritin <30 ng/mL, which is defined as within the range of insufficiency to deficiency [16,17]. Data in this patient age group also suggest that IDA is associated with low birth weight, low dietary iron intake, low household income, and long duration of breastfeeding [17,18,19].

Because feeding is the primary source of iron intake for infants, feeding practices considerably impact their iron status. Infant feeding practices during the first 6 months of life vary widely. Some infants are exclusively breastfed, whereas others are given infant formula or a combination of both breast milk and formula. Some infants may also be given complementary foods early before they have reached 6 months of age. Although iron supplementation for breastfed infants, as recommended in the AAP guideline, is not endorsed by Thai health organizations, some clinical practitioners in Thailand adhere to this practice because iron supplementation may have a preventive effect on ID in exclusively or partially breastfed infants.

In this study, we investigated the association of infant feeding practices with iron status and hematologic parameters among infants at 6 months of age using historical data of patients examined in a well-baby clinic. Our primary objective was to compare the prevalence of anemia, ID, and IDA at 6 months of age between infants who received different types of feeding (breast milk only, breast milk with iron supplements, a combination of breast milk and infant formula with or without iron supplements, and infant formula only). The secondary objective was to identify possible factors associated with IDA at 6 months of age in these infants.

## 2. Methods

We performed a retrospective cohort study and reviewed the electronic medical records of healthy infants visiting the well-baby clinic at Chakri Naruebodindra Medical Institute (CNMI), Samut Prakan, Thailand, from 1 January 2019 to 30 December 2020. This study was approved by the Faculty of Medicine Ramathibodi Hospital, Mahidol University Ethics Committee (register No. MURA2021/44). The study was performed in accordance with the International Ethical Guidelines for Biomedical Research Involving Human Subjects and ethical principles of the Declaration of Helsinki. A waiver of individual patient informed consent was granted.

During the study period, a protocol for universal anemia screening in 6-month-old infants was implemented in the well-baby clinic at CNMI, including complete blood count (CBC) and serum ferritin measurement. All patients with results of serum ferritin measurement performed during the study period were identified in the CNMI laboratory electronic database. Only infants aged 5–7 months with CBC and serum ferritin results were selected for chart review. Only the medical records of infants with available information from 4-month and 6-month well-baby visits in the electronic database were included in the study. Premature infants with gestational age at birth less than 35 weeks and infants who received iron supplements before 4 months of age were excluded from the study.

A self-administered questionnaire is routinely provided to the parents of infants visiting a well-baby clinic at CNMI, to be completed before each physician encounter. These questionnaires, which are tailored according to infants’ age (2 months, 4 months, or 6 months), specifically query the parents about current infant feeding practices and developmental milestones, as well as parents’ general knowledge about child-rearing. Infant weight, length, and head circumference measurements are performed by skilled nurses at the clinic before each physician encounter. During each visit with a pediatrician, the infant’s anthropometric data, together with a patient history and findings of physical examination performed by the pediatrician, are recorded in the form of a physician’s note. At the end of each visit, the questionnaire and physician’s note are entered into the electronic medical records database. Data extraction was performed using these documents. Data collection consisted of infants’ baseline characteristics, including sex, gestational age, weight, length, and head circumference at birth, and the data from continuous infant monitoring at the well-baby clinic at 4 and 6 months of age, including weight, length, head circumference, and infant feeding practice. We collected laboratory results, including CBC and serum ferritin levels at 6 months of age. Infants were categorized into four groups according to feeding practices during the 4 to 6-month visits: (1) breastfed infants (BF) were those who were fed breast milk (without infant formula or iron supplements); (2) breastfed with iron supplements (BI) were those who were fed breast milk and also received iron supplements prescribed by a physician starting at the 4-month visit; (3) mixed-fed with or without iron supplements (MF) were those who were exposed to both breast milk and infant formula (infants in this group may or may not have received iron supplementation); and (4) formula-fed infants (FF) were those who were fed infant formula (without breast milk or iron supplementation). There were 2 pediatric iron supplement products available in the hospital at the time of the study; ferrous fumarate suspension with 15 mg of elemental iron per 0.6 mL, and iron (III) hydroxide polymaltose complex syrup with 10 mg of elemental iron per 1 mL. All infants who received iron supplementation were prescribed either of these products at the dose of 1–2 mg/kg/day of elemental iron.

### 2.1. Biochemical Analyses

CBCs were conducted using an automated hematology analyzer (Sysmex XN 3000; Sysmex Asia Pacific Pte Ltd., Jalan Kilang, Singapore). Serum ferritin concentrations were measured using a sandwich-type electrochemiluminescence immune assay (Cobas 6000; Roche Diagnostics, Basel, Switzerland), with a measurement range of 0.5–2000 ng/mL. Internal quality control runs were performed daily, per manufacturer guidelines. All external quality assessment was performed according to the Bio-Rad Laboratories Ltd. EQAS program, accredited to ISO 17043:2010. In this study, iron deficiency (ID) in infants was defined as serum ferritin <12 ng/mL, anemia in infants was defined as Hb < 11 g/dL, and iron deficiency anemia (IDA) in infants was defined as both serum ferritin <12 ng/mL and Hb < 11 g/dL [20].

### 2.2. Statistical Analysis

We performed univariate analyses to identify significant differences between the groups. One-way analysis of variance and post-hoc Bonferroni tests were used for parametric continuous variables, and results are presented as mean ± standard deviation. Kruskal-Wallis and post-hoc Mann–Whitney U tests were used for non-parametric continuous variables. Results are presented as median (interquartile range). Pearson’s chi-square or Fisher exact tests were used for categorical variables (pre-hoc and post-hoc pairwise analysis), and the results are presented as number (%). To determine the factors associated with IDA in infants at 6 months old, selected infant’s baseline characteristics (gestational age, birth weight, sex, small gestational age, weight gain during the first 6 months of life) and infant’s feeding habits (complementary feeding initiated before 6 months, and feeding practices) were used as variables. Then, a uni-variate logistic regression was performed to investigate the simple association between each variable with infant IDA. Factors demonstrating an association with *p* < 0.1 in univariate logistic regression were entered into the multivariate logistic regression model. When feeding practices were included in the univariate and multivariate logistic regression analysis, the BF group was set as the reference group. A *p*-value < 0.05 was considered statistically significant. However, the Bonferroni-adjusted *p*-value for significance of 0.008 was set for post-hoc (pairwise) analysis of categorical variables. Statistical analyses were performed using SPSS version 18.0 (SPSS Inc., Chicago, IL, USA).

## 3. Results

**Figure 1:**
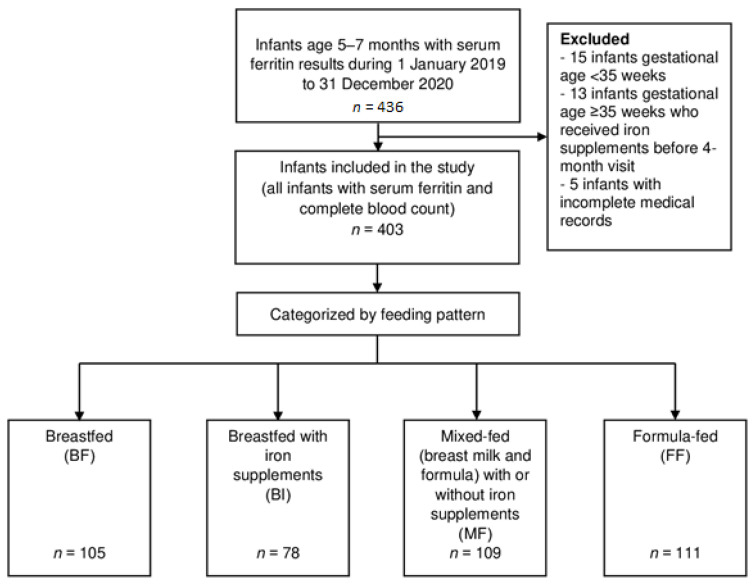
Flow chart of infants in the study.

During the study period, 403 infants met the inclusion criteria and were enrolled in this study. The infants were categorized according to four feeding patterns, as follows: 105 infants in the BF group, 78 infants in the BI group, 109 infants in the MF group, and 111 infants in the FF group. Figure 1 shows the number of study participants and the reasons for exclusion.

There were no significant differences in the infants’ baseline characteristics between the four groups, other than infants categorized as large for gestational age. Complementary feeding was introduced before 6 months of age in 31.1% of BF, 53.1% of BI, 79.8% of MF, and 85.7% of FF infants. All infants (100%) in the BI group and 40 (36.7%) in the MF group received iron supplements, started at the 4-month visit, whereas no infants in the BF and FF groups received iron supplements (Table 1).

The mean age of infants at the time of laboratory evaluation was 189 ± 11 days in BF, 190 ± 11 days in BI, 192 ± 11 days in MF, and 193 ± 9 days in FF infants, respectively. Laboratory findings at 6 months showed that infants in the BF group had significantly lower mean serum ferritin levels, Hb, HCT, mean corpuscular volume (MCV), mean corpuscular hemoglobin (MCH), and mean corpuscular hemoglobin concentration (MCHC) in comparison with the other groups. In contrast, the mean red blood cell distribution width (RDW) of infants in the BF group was significantly higher than that in the other groups.

At 6 months of age, 38.1% of infants in the BF group had anemia, 28.6% had ID, and 17.1% had IDA, which were all significantly higher than these proportions in the other groups. In contrast, there was no significant difference in the rate of infants who had anemia without ID between the four groups (Table 2).

Univariate logistic regression analysis was performed to determine the predictive factors for IDA in 6-month-old infants. Male sex and greater weight gain during 0–6 months were associated with an increased risk of IDA whereas older gestational age and higher birth weight were associated with a decreased risk of IDA. The BI, MF, and FF groups were associated with a decreased risk of IDA, with BF infants as the reference group. In contrast, small for gestational age and complementary feeding initiated before 6 months were not associated with the risk of IDA. In the multivariate logistic regression model, which included factors with *p* < 0.1 in univariate logistic regression analysis, greater weight gain during 0–6 months slightly increased the risk of IDA and higher birth weight slightly decreased this risk. Infant feeding patterns strongly predicted IDA in the multivariate logistic regression model. With BF infants as the reference group, the BI, MF, and FF groups had a 90.4%, 97.5%, and 96.9% decreased risk of IDA, respectively (Table 3).

## 4. Discussion

Our study found that 28.6% and 17.1% of Thai infants who were breastfed but did not receive iron supplementation had ID and IDA, respectively, at 6 months of age. These incidences were significantly higher than those among formula-fed or partially breastfed infants. We found that breastfed infants who received 1–2 mg/kg/day of iron supplementation starting at 4 months of age had iron status and hematologic parameters comparable to those of formula-fed or partially breastfed infants. Infants in the BF group had significantly lower mean serum ferritin levels and significant differences in hematologic parameters compatible with IDA (low Hb, HCT, MCV, MCH, MCHC, and high RDW) in comparison with infants with other feeding patterns.

When infants in all groups were combined, the overall prevalence of anemia in 6-month-old infants was 23.9%, which is comparable to the prevalence previously reported in multiple studies among infants and children in similar age groups in Thailand. A study by Suwannakeeree et al. found a 29.1% prevalence of anemia in 9-month-old infants [19], and Tantracheewathorn et al. found a 26.4% prevalence in 9–12 month-old infants [18]. Rojroongwasinkul et al. reported a 26.0% prevalence in urban children age between 0.5 to 2.9 years in the South East Asian Nutrition Survey [21]. The overall prevalence of IDA among infants in our study was 5.5%, which was lower than the previously reported prevalence among 9–12 month-old infants in Thailand (14.3% by Tantracheewathorn et al. [18] and 17.9% by Suwannakeeree et al. [19], and prevalence among 6–12 month-old infants in other countries (6.6% in Taiwan [22] and 6.9% in New Zealand [23]. This difference in IDA prevalence was likely owing to differences in the ages of the study populations. The iron store at birth is the source for iron utilization in infants until approximately 6 months of age. The risk of IDA among infants over 6 months old is increased unless appropriate complementary feeding is given. Additionally, the feeding patterns differed between studies. Our study included a large number of formula-fed infants, many of whom were breastfed or partially breastfed and receiving iron supplements beginning at 4 months old, which may help to prevent IDA.

In our study, 15–20% of infants in each group were anemic despite having normal serum ferritin levels at 6 months. The mean MCV of 74, non-ID anemic infants was 68.1 ± 8.0 fL, lower than the mean MCV in every feeding group. Anemia among these infants was unlikely to have been caused by ID. Most anemic infants in our study with serum ferritin levels above the cut-point may have thalassemia and hemoglobinopathy genes because thalassemia is endemic in Thailand. A study among healthy infants aged 6–12 months in the northern part of Thailand showed that 29.4% of infants were thalassemia carriers and 2.4% had thalassemia disease [24]. Other nutritional deficiencies, such as vitamin A, vitamin B12, and folate, or parasitic diseases or infestation such as malaria or hookworm, could also contribute to anemia in infants [25]. Unfortunately, owing to the lack of consistency in patient management, follow-up, and further laboratory investigation, the cause of anemia in these infants could not be confirmed.

Previous studies have revealed evidence linking exclusive breastfeeding during the first 6 months of life or longer to an increased risk of IDA among infants at 6–12 months of age [17,18,19,22]. Current practice guidelines from the Ministry of Health of Thailand recommend weekly iron supplements (12.5 mg of elemental iron once a week) for infants starting at 6 months of age [26]. This recommendation can ensure the iron status of infants older than 6 months of age, but the adequacy of iron for infants during the first 4–6 months of life remains unclear. As demonstrated in our study, approximately one in every four breastfed infants had ID at 6 months of age, and nearly one in every five breastfed infants had IDA. Given the potential negative effects of even pre-anemic ID on early development, waiting until infants reach 6 months of age before starting supplementation may be too late. A meta-analysis of iron supplementation among breastfed infants at an early age showed limited evidence regarding the effect of iron supplements in the prevention of IDA [27]. However, the impact of supplements depends on the initial infant iron status, as shown in a randomized control trial that compared the prevalence of IDA among exclusively breastfed infants with iron supplementation during ages 4–9 and 6–9 months. That study was conducted in two areas (Honduras and Sweden) with relatively different amounts of dietary iron consumption. Iron supplementation in breastfed infants from 4 months effectively reduced the prevalence of ID at 6 months of age only in Honduras, where the baseline prevalence of ID is high and dietary iron intake is low [28]. Neonatal iron storage has a more significant effect on serum ferritin levels in early infancy than iron consumption from breast milk [29]. Our study findings imply that a considerable number of breastfed infants who attend our clinic may have low iron stores at birth, which are affected by maternal nutrition and dietary intake during pregnancy. Iron supplements starting at 4 months of age in breastfed infants, as recommended by the AAP, can offer a reasonable approach to reduce the risk of ID and IDA among Thai infants.

Our results showed a significantly lower rate of ID and IDA among breastfed infants with iron supplementation, as well as those who were partially breastfed and formula-fed, compared with infants who were breastfed and did not receive iron supplements. Our multivariate logistic regression analysis confirmed these findings and showed that iron supplementation among breastfed infants might reduce the risk of IDA by 90.4%. In addition to iron supplements, evidence from a systematic review suggests that the introduction of solids at 4 months may have a beneficial effect on the rate of IDA in breastfed infants [30]. In our study, complementary foods were introduced to infants in each group between age 4 and 6 months at a different rate. However, complementary feeding did not seem to affect iron status, which showed no association with IDA in univariate analysis (Table 3). The absence of an association between complementary feeding and IDA may be owing to feeding practices in Thailand, where typical foods given to infants at this age are usually plant-based foods with low iron content [31]. As a retrospective study, we did not have information regarding the types of foods given to infants.

Universal iron supplementation of breastfed infants at an early age is controversial. Iron homeostasis in infants younger than age 6 months is limited compared with older infants [32]. Iron absorption is not effectively down-regulated in infants at an early age, who have adequate body iron; this raises concerns about iron toxicity or other side effects of iron supplementation among iron-sufficient infants. A systematic review and meta-analysis in 2013 found that daily iron supplementation impaired length gain and weight gain over the follow-up period, despite no differences in final weight or length [33]. Despite some concerns regarding infants’ growth with daily iron supplementation, we found no significant differences in growth parameters among infants with different feeding patterns. However, our study included a relatively small sample size, and follow-up assessment was performed over a short period. Thus, we cannot support nor refute the above concerns. Adequately powered trials are needed to investigate the non-hematological benefits and risks of iron supplementation in this patient population. As an alternative to iron supplementation, measures to increase iron stores at birth among infants so as to provide them with adequate iron until late infancy, such as delayed cord clamping, have been reported [34].

This study has some limitations. Our study was performed at a single center with a limited sample size, so it was not sufficiently powered to be able to adequately assess the non-hematological effects of different feeding interventions. This study used retrospective data collected from electronic medical records; maternal baseline information during pregnancy and delivery as well as details regarding complementary foods given to infants were lacking. Information about feeding practices was based solely on parents’ responses to questionnaires and interviews, which could have resulted in recall bias. Compliance with prescribed iron supplements was difficult to assess and could have resulted in misclassification bias. In our study, a simultaneous measurement of C-reactive protein to rule out infection was not performed at the time of laboratory serum ferritin measurement, as recommended by the AAP [14]. An elevated serum ferritin level in some infants in our study might indicate conditions such as inflammation, infection, malignancy, or liver disease rather than total body iron level. However, it is unlikely that infants had any of these conditions at well-child health maintenance visits, when laboratory testing was performed. Although serum ferritin is a good indicator of iron storage, there are a few other indicators that could be more accurate. Most participants in this study lived in the Samut Prakan Province, which is located on the outskirts of Bangkok. Therefore, the data may not be representative of populations in other areas of Thailand.

## 5. Conclusions

Anemia is not uncommon among Thai infants at 6 months of age. In our study, a significantly higher incidence of anemia, ID and IDA was found in Thai BF than FF or MF infants. However, iron supplements (1–2 mg/kg/day) in BF infants starting at 4 months significantly reduced their ID and IDA incidence.

## Figures and Tables

**Table 1:** Baseline characteristics of infants in the study.

Characteristics	Breastfed (BF) (*n* = 105)	Breastfed with Iron Supplements (BI) (*n* = 78)	Mixed-Fed with or without Iron Supplements (MF) (*n* = 109)	Formula-Fed (FF) (*n* = 111)	*p*-Value
Male sex	57 (54.3)	37 (47.4)	59 (54.1)	52 (46.9)	0.564
Inborn	43 (41.0)	33 (42.3)	49 (45.0)	42 (37.8)	0.757
Gestational age (weeks)	38 ± 1	38 ± 1	38 ± 1	38 ± 1	0.521
Small for gestational age	13 (12.4)	18 (23.1)	12 (11.0)	15 (13.5)	0.127
Large for gestational age	0 (0)	3 (3.9)	0 (0)	5 (4.5)	0.008
At birth					
Weight (g)	3103 ± 391	3105 ± 461	3120 ± 397	3195 ± 449	0.251
Length (cm)	49.5 ± 2.2	49.2 ± 2.1	49.6 ± 2.2	50.1 ± 2.2	0.971
Head circumference (cm)	33.6 ± 1.4	33.7 ± 1.4	33.7 ± 1.2	34.0 ± 1.4	0.411
At 4-month visit					
Weight (g)	6606 ± 813	6569 ± 905	6598 ± 895	6727 ± 842	0.699
Length (cm)	62.8 ± 2.2	62.7 ± 2.5	63.0 ± 2.3	63.4 ± 2.2	0.681
Head circumference (cm)	41.0 ± 1.2	40.8 ± 1.4	41.0 ± 1.4	40.9 ± 1.3	0.675
At 6-month visit					
Weight (g)	7468 ± 853	7475 ± 1065	7603 ± 1007	7730 ± 924	0.158
Length (cm)	66.1 ± 2.3	66.2 ± 2.6	66.8 ± 2.3	67.3 ± 2.4	0.523
Head circumference (cm)	42.7 ± 1.3	42.5 ± 1.4	42.7 ± 1.5	42.6 ± 1.2	0.327
Complementary feeding before 6 months	36 (34.3)	41 (52.6)	87 (79.8)	95 (85.6)	<0.001
Iron supplementation	0 (0)	78 (100.0)	40 (36.7)	0 (0)	<0.001

Values in the table are *n* (%) or mean ± standard deviation.

**Table 2:** Laboratory characteristics of infants categorized by feeding practices.

Characteristics	Breastfed (BF) (*n* = 105)	Breastfed with Iron Supplements (BI) (*n* = 78)	Mixed-Fed with or without Iron Supplements (MF) (*n* = 109)	Formula-Fed (FF) (*n* = 111)	*p*-Value
Hb (g/dL)	11.1 ± 1.0 ^a^	11.7 ± 1.0	11.8 ± 0.9	11.8 ± 0.9	<0.001
HCT (%)	34.4 ± 2.9 ^b^	36.2 ± 3.0	36.2 ± 2.6	35.3 ± 2.6	<0.001
MCV (fL)	69.8 ± 6.9 ^c^	71.8 ± 7.1	73.7 ± 6.5	74.3 ± 6.0	<0.001
MCH (pg)	22.6 ± 2.5 ^c^	23.4 ± 2.7 ^e^	24.1 ± 2.4	24.8 ± 2.2	<0.001
MCHC (g/dL)	32.3 ± 1.1 ^e^	32.5 ± 1.0 ^e^	32.7 ± 1.1 ^e^	33.3 ± 1.0	<0.001
RDW (%)	14.6 ± 2.3 ^e^	14.5 ± 2.2 ^e^	14.0 ± 2.1	13.4 ± 1.9	<0.001
Serum ferritin (ng/mL)	37.5 ± 43.4 ^a^	88.7 ± 84.1	74.8 ± 53.6	80.2 ± 47.6	<0.001
Anemia (Hb < 11 g/dL)	40 (38.1) ^c,†^	17 (21.8)	21 (19.3)	18 (16.2)	<0.001
ID (Serum ferritin < 12 ng/mL)	30 (28.6) ^a,†^	3 (3.8)	4 (3.7)	1 (0.9)	<0.001
IDA	18 (17.1) ^a,†^	2 (2.6)	1 (0.9)	1 (0.9)	<0.001
ID without anemia	12 (11.4) ^d,^^†^	1 (1.3)	3 (2.8)	0 (0)	<0.001
Anemia without ID	22 (21.0)	15 (19.2)	20 (18.3)	17 (15.3)	0.754

Values in the table are *n* (%) or mean ± standard deviation. a—significant difference with BI, MF, FF; b—significant difference with BI, MF; c—significant difference with MF, FF; d—significant difference with BI, FF; e—significant difference with FF. †—significant difference with *p*-value < 0.008. Hb—hemoglobin, HCT—hematocrit, ID—iron deficiency, IDA—iron deficiency anemia, MCV—mean corpuscular volume, MCH—mean corpuscular hemoglobin, MCHC—mean corpuscular hemoglobin concentration, RDW—red blood cell distribution width.

**Table 3:** Logistic regression analyses of factors associated with iron deficiency anemia in 6-month-old infants.

Variables	Crude OR	95% CI	*p*-Value	Adjusted OR	95% CI	*p*-Value
Gestational age	0.586	0.392–0.875	0.009	0.819	0.464–1.447	0.819
Birth weight	0.998	0.997–0.999	0.003	0.997	0.995–0.999	0.002
Male sex	2.709	1.038–7.072	0.042	2.585	0.818–8.168	0.106
Weight gain 0–6 months	1.001	1.000–1.001	0.011	1.001	1.000–1.001	0.010
Small for gestational age	1.346	0.439–4.128	0.604			
Complementary feeding before 6 months	0.651	0.274–1.546	0.331			
Feeding practices						
BF	1 (ref)			1 (ref)		
BI	0.127	0.029–0.566	0.007	0.096	0.020–0.466	0.004
MF	0.045	0.006–0.342	0.003	0.025	0.004–0.267	<0.001
FF	0.044	0.006–0.336	0.003	0.031	0.003–0.218	0.002

BF—breastfed, BI—breastfed with iron supplements, MF—mixed-fed with or without iron supplements, FF—formula-fed, OR—odds ratio, CI—confidence interval.

## Data Availability

The datasets used and/or analysed during the current study are available from the corresponding author on reasonable request.

## Abbreviations

Hb	hemoglobin
HCT	hematocrit
ID	iron deficiency
IDA	iron deficiency anemia
MCH	mean corpuscular hemoglobin
MCHC	mean corpuscular hemoglobin concentration
MCV	mean corpuscular volume
RDW	red blood cell distribution width
